# High Association between Human Circulating MicroRNA-497 and Acute Myocardial Infarction

**DOI:** 10.1155/2014/931845

**Published:** 2014-07-07

**Authors:** Zhenci Li, Jing Lu, Yi Luo, Shaonan Li, Minsheng Chen

**Affiliations:** ^1^Department of Cardiology, Guangzhou First Municipal People's Hospital, Guangzhou Medical University, Panfu Road 1, Guangzhou, Guangdong 510180, China; ^2^Southern Medical University, Guangzhou Avenue North 1838, Guangzhou, Guangdong 510515, China

## Abstract

Recent papers have reported the fundamental roles of miR-497 in infarction which acute myocardial infarction (AMI) belongs to. However, the expression levels of miR-497 in AMI patients were unclear, especially the circulating miR-497 that was detectable in the human plasma. In this study, we focused on the expression levels of circulating miR-497 in AMI and the roles of plasma miR-497 as a promising biomarker for AMI. The plasma miR-497 levels were detected from 27 AMI patients and 31 healthy volunteers by qRT-PCR. The cTnI concentrations of these samples were also analyzed by ELISA. Results showed circulating miR-497 levels were upregulated in AMI patients at 4 h, 8 h, 12 h, and 24 h, by contrast to those in control. Interestingly, time courses of circulating miR-497 levels displayed similar trends to that of cTnI concentrations in AMI patients; further study revealed the high correlation between circulating miR-497 and cTnI concentrations (*r* = 0.573, *P* < 0.001). At last, the receiver operating characteristic (ROC) curve was performed and declared that there was a faithworthy sensitivity and specificity to identify the AMI patients by using circulating miR-497. In conclusion, circulating miR-497 might be a promising biomarker for AMI identification and there was high association between human miR-497 and acute myocardial infarction.

## 1. Introduction

Acute myocardial infarction (AMI), one of the most serious cardiovascular diseases, remains a leading cause of morbidity and mortality worldwide [1]. Rapid and precise diagnosis provides opportunity to immediate therapy and to dramatically decrease the mortality. However, it is straightforward, difficult, or somewhere in between to recognize if the patient is suffering from AMI [2]. At present, electrocardiography (ECG) and echocardiography were applied to identify the patient whose clinical picture raised a suspicion of AMI. Besides these, some biomarkers in the blood have been performed to diagnose the AMI, also, such as cardiac troponin I (cTnI) and creatine kinase-MB which were denoted as real value of biomarkers [3]. However, new biomarkers that can improve current diagnosis for AMI are still lacking. Recent studies implied that circulating myocardial-derived microRNAs (miRNAs) possessed the ability as potential biomarkers for diagnosis of AMI [4–6].

miRNAs, composed of about 22 nucleotides, regulate about 60% gene expression by targeting the 3′-untranslated regions (3′-UTRs) to result in negative modulation of relevant mRNAs for expression [7]. The miRNAs play versatile roles in cellular biology and pathophysiologic regulatory pathways [8, 9]. In terms of cardiovascular system, miRNAs have been documented to act as regulators in vessel formation, angiogenesis, and cardiac development, in which depletion of functions of miRNAs results in defects [10–12]. It is found that dysregulation of miRNAs has been involved in cardiac disease. Recent reports have revealed that miRNAs are detectable in serum, plasma, urine, and other body fluids [6, 13–15]. Up to present, some circulating miRNAs were investigated and considered as unique biomarkers for diagnostic and therapeutic interventions of AMI, for example, miR-208, miR-499, miR-133, and miR-1 [16, 17].

The study on regulatory mechanism of miR-497 mainly refers to be a potential prognostic marker and functions as a tumor suppressor in human cervical cancer, breast cancer, and hepatocellular carcinoma [18–20]. Interestingly, it is noteworthy that miR-497 has emerged as key mediators of posttranscriptional gene silencing in both pathogenic and pathological aspects of ischemic stroke biology [21]. Moreover, Yin et al. found that knockdown of miR-497 effectively attenuates ischemic brain infarction and improves neurological outcomes in mice after focal cerebral ischemia [22]. So it is suspected that miR-497 takes fundamental effects on infarction that AMI belongs to. However, the expression levels of miR-497 in AMI were unclear, especially the circulating miR-497 that was detectable in the human plasma [23].

In this study, we focused on the expression levels of circulating miR-497 in AMI and the roles of plasma miR-497 as a promising biomarker for AMI.

## 2. Materials and Methods

### 2.1. Human Blood Samples

Human beings study has been approved by the Ethics Committee, Ministry of Health, China, and informed consents were written in this work. Blood samples were obtained from Guangzhou First Municipal People's Hospital and Nan Fang Hospital. 27 Patients with AMI were diagnosed according to several criteria: (i) ischemic symptoms; (ii) increased cardiac cTnI level; (iii) creatine kinase-MB (CK-MB); (iv) pathological Q wave; and (v) ST-segment elevation or depression [24]. 31 healthy volunteers with normal electrocardiogram and no history of cardiovascular were recruited as the control group. Plasma samples of AMI patients were acquired at 4 h (±30 min), 8 h (±30 min), 12 h (±30 min), 24 h (±60 min), 48 h (±60 min), and 72 h (±60 min) once the onset of symptoms occurred. Plasma samples were stored at −70°C before RNA was extracted.

### 2.2. Measurement of Plasma Cardiac Troponin I

Plasma cTnI concentrations were detected by ELISA assay according to manufacturer's protocol (Abnova).

### 2.3. Quantitative Real-Time PCR

Plasma miR-497 was isolated by using miRNeasy Mini Kit (Qiagen, Valencia, CA). Reverse transcription was performed using the TaqMan miRNA reverse transcription kit (Takara, Japan). qRT-PCR was performed on ABI 7500 with SYBR Premix Ex Taq Kit (Takara, Japan). In short, the reactions were started at 95°C for 30s, followed by 40 cycles of 95°C for 15 sec and 60°C for 1 min. Internal control was the U6 small nuclear RNA. Ct value of samples was recorded. Data were normalized by using 2^−ΔΔCt^ method [25].

### 2.4. Data Analysis and Statistics

The data of miR-497 and cTnI were analyzed by the Kolmogorov-Smirnov test to examine whether they followed the normal distribution. Comparisons of two groups were analyzed by Student's *t*-test and the differences of three groups (or more than three) were compared by ANOVA once the data are in normal distribution. For categorical variables, the chi-square test was used. The correlation was analyzed by linear regression analysis. The receiver operating characteristic (ROC) curve was performed and the area under the ROC curve (AUC) was calculated. Leave-one-out cross-validation with a logistic regression model was used to simulate the performance of a classification algorithm [26]. Statistical analyses were conducted by SPSS 17.0 software. Statistically significant was shown as *P < 0.05 and **P < 0.01.

## 3. Results

### 3.1. Statistical Analysis of Clinical Characteristics

To study if there was association between clinical characteristics and AMI and to eliminate the confounders from samples selection, 27 patients with AMI and 31 healthy volunteers were enrolled. All AMI patients were diagnosed based on combination of several criteria as shown in Materials and Methods and had transmural AMI. The details of clinical characteristics were displayed in Table 1. Age, gender, white blood cell, systolic blood pressure, diastolic blood pressure, glucose, triglyceride, total cholesterol, high-density lipoprotein, low-density lipoprotein, diabetes mellitus, creatinine, hyperlipidaemia, and hypertension were compared to analyze the differences separately. We did not find significant statistical differences in these clinical characteristics between AMI patients and healthy volunteers (*P* > 0.05). This illustrated that the bias from patient selection was eliminated.

### 3.2. Comparison of Circulating miR-497 and cTnI Levels in Patients with Those in Healthy Volunteers

**Figure 1:**
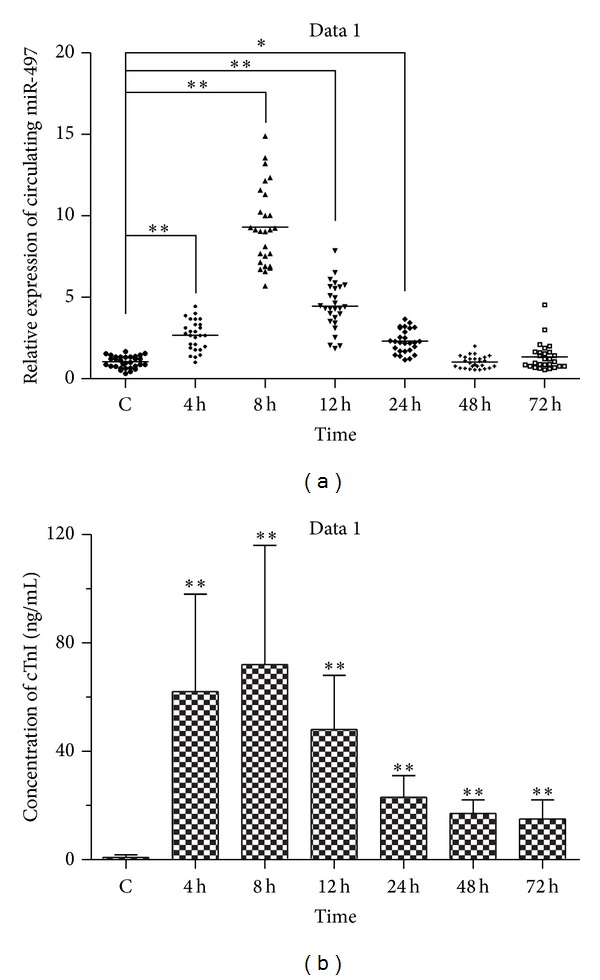
Expression of circulating miR-497 and concentrations of cTnI in AMI patients. The relative expression of circulating miR-497 in AMI patients was assayed at 4 h, 8 h, 12 h, 24 h, 48 h, and 72 h, compared with that in healthy adults (a). Concentrations of cTnI were also detected at the same time point (b). Data were denoted as mean ± SEM. Statistically significant was shown as *P < 0.05 and **P < 0.01.

We assessed the expression of circulating miR-497 in AMI patients at 4 h (±30 min), 8 h (±30 min), 12 h (±30 min), 24 h (±30 min), 48 h (±30 min), and 72 h (±30 min), by contrast to that in healthy volunteers, by using qRT-PCR. Δct levels of miR-497 were −5.23 ± 0.67, −3.99 ± 1.47, −4.62 ± 1.35, −5, 62 ± 1.68, −6.22 ± 0.86, and −6.34 ± 1.15 at 4 h, 8 h, 12 h, 24 h, 48 h, and 72 h, respectively. It showed that expression of miR-497 was enhanced significantly at 4 h, 8 h, 12 h, and 24 h as shown in Table 2 and Figure 1(a). There was no significant difference of expression of miR-497 at 48 h and 72 h. Meanwhile, the circulating cTnI concentrations were also assayed by ELISA assay. miR-497 and cTnI were measured from the same samples at same time points. cTnI peak (76.24 ng/mL mean) was reached at 8 h in AMI patients over control. cTnI levels in AMI patients increased significantly at 4 h, 8 h, 12 h, 24 h, 48 h, and 72 h, respectively, compared with control (*P* < 0.001) (Figure 1(b)).

### 3.3. Analysis of Correlation between Circulating miR-497 Levels and AMI

**Figure 2:**
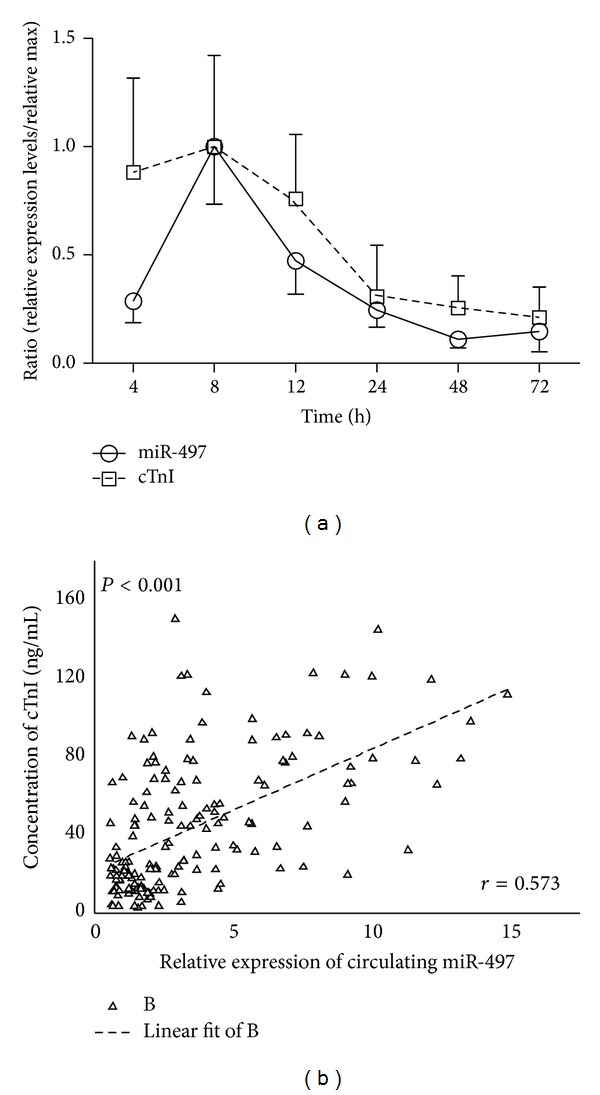
Correlation between circulating miR-497 and plasma cTnI. Time courses of circulating miR-497 and cTnI in AMI patients (a). The correlation between miR-497 and cTnI in AMI patients by using linear regression analysis (b). Data were presented as mean ± SEM, *P < 0.05, and **P < 0.01.

Moreover, time courses were depicted to analyze the dynamic process of circulating miR-497 and cTnI levels in AMI patients. It showed that the miR-497 levels were changed in a time dependent manner. The miR-497 levels reached the peak at 8 h and were reduced gradually after that time point. The cTnI time courses displayed the same trends (Figure 2(a)). Furthermore correlation analysis was conducted to analyze the correlation between circulating miR-497 levels and cTnI. The data showed a positive correlation between circulating levels of miR-497 and cTnI concentrations in AMI patients (*r* = 0.573, *P* < 0.001) (Figure 2(b)). These data suggested that circulating miR-497 may be considered as a novel biomarker of AMI.

### 3.4. Specificity and Sensitivity of Circulating miR-497

**Figure 3:**
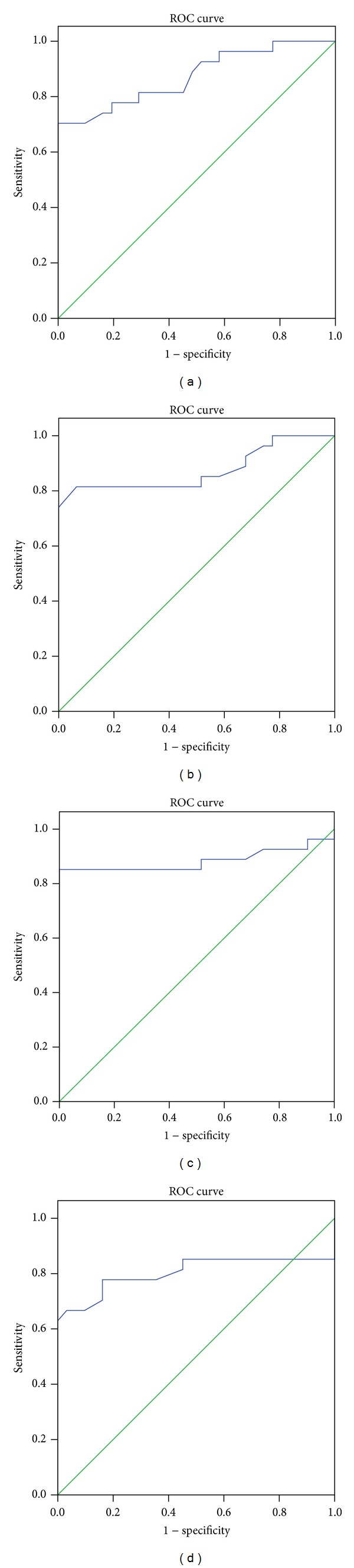
Diagnostic value of circulating miR-497 at different time points. ROC curve analyzed the diagnostic value of circulating miR-497 at 4 h (a), 8 h (b), 12 h (c), and 24 h (d) for AMI identification from healthy group.

ROC curve with AUC was performed by using miR-497 levels to differentiate the AMI group from the healthy group (Figure 3). The AUC was 0.87, 0.88, 0.88, and 0.81 (Figure 3 and Table 3) at 4 h, 8 h, 12 h, and 24 h. By the threshold score of 1.52, 1.55, 1.61, and 1.37 above that patients are predicted as the AMI patients, we gained a specificity of 90%, 94%, 95%, and 84% and a sensitivity of 81%, 82%, 85%, and 80% for the identification of AMI patients. To detect if the defined threshold was overfitted to the data, leave-one-out cross-validation with a univariate logistic regression classifier was performed to differentiate the AMI group from healthy group. This data produced a sensitivity of a specificity of 93%, 95%, 94%, and 88% and a sensitivity of 82%, 86%, 85%, and 80% which are similar to the above result. This demonstrated that overfitting of threshold was minimal. These results declared that there was faithworthy sensitivity and specificity to identify the AMI patients by using circulating miR-497.

## 4. Discussion

miRNAs are detectable in peripheral circulation and are highly stable in boiling water and in solution with very high or low pH. Moreover, circulating miRNAs exhibit the ability of being resistant to plasma RNase activity [27, 28]. The performance of miRNAs as biomarkers has been shown in diagnosis and monitoring of human diseases, such as lung cancer, breast cancer, gastrointestinal cancer, and other solid tumors [29]. Apart from these, miRNAs also take highly promising effects on diagnosis and monitoring of infectious diseases, for example, the active tuberculosis, viral hepatitis, and hand-foot-and-mouth disease [30–32]. The most important is the fact that there have been some miRNAs reported as the potentially promising biomarkers for diagnosis of AMI [16, 17, 29]. All these reports explored the roles of circulating miRNAs in investigation of human disease, especially in AMI. Because of fundamental effects of miR-497 on infarction [22], we made the question if the miR-497 was associated with AMI which belongs to the infarction and if circulating miR-497 could be denoted as promising biomarker for diagnosing AMI.

In this study, we compared the expression levels of circulating miR-497 in AMI with that in healthy volunteers. Interestingly, results displayed that the circulating miR-497 levels were overexpressed at 4 h, 8 h, 12 h, and 24 h in patients, contrasted by that in healthy volunteers as shown in Figure 1(a) and Table 2. This data supported our previous hypothesis and demonstrated that the miR-497 was highly associated with human AMI indeed, notwithstanding the exact regulatory mechanism of miR-497 was unclear. It is the first report on the roles of miR-497 on human AMI.

To identify if the circulating miR-497 exhibited the promising roles as a biomarker for diagnosing AMI, we detected the plasma concentrations of cTnI, a classic marker of myocardial injury (Figure 1(b)), and assessed the correlation between plasma cTnI and miR-497. The time course curve of plasma cTnI concentrations was similar to miR-497 (Figure 2(a)). Further study supported the correlation between plasma cTnI and miR-497 by statistical analysis (Figure 2(b) *r* = 0.573, *P* < 0.001). It illustrated that circulating miR-497 may have potential performances for diagnosing the AMI, just like the plasma cTnI [3, 33].

In our study, we choose the clinical samples with similar age, gender, white blood cell, systolic blood pressure, diastolic blood pressure, glucose, triglyceride, total cholesterol, high-density lipoprotein, low-density lipoprotein, diabetes mellitus, creatinine, hyperlipidaemia, and hypertension to eliminate the confounders from samples selection. According to statistical analyses, the data manifested that there was no correlation between plasma miR-497 levels and the clinical characteristics (Table 1). It also suggested the specific roles of plasma miR-497 levels in diagnosing AMI.

At last, ROC curve of miR-497 was plotted to investigate the informativeness for AMI diagnosis. We acquired an AUC of 0.87, 0.88, 0.88, and 0.81 at 4 h, 8 h, 12 h, and 24 h, individually (Figure 3 and Table 3). By using denoted threshold score of circulating miR-497, the high specificity and sensitivity were achieved at 4 h, 8 h, 12 h, and 24 h for detection of AMI from control group. So, the data revealed that the circulating miR-497 had the effective performance for AMI diagnosis and supported the previous presumption that circulating miR-497 was effective to identify the AMI as a novel biomarker.

Some dysregulated miRNAs are documented to be involved in cell differentiation, hypoxia, fibrosis, and development in response to AMI [34, 35]. It suggested that miRNAs played critical roles in AMI pathophysiological processes. miRNAs are endogenous regulators of gene expression. Thus, it is reasonable to make a hypothesis that miR-497 could be highly associated with the regulation of AMI pathophysiological processes, which was not reported up to date. A relatively small number of samples was enrolled to prove the consideration of circulating miR-497 as a biomarker for AMI in this study. Larger number of clinical samples studies should be required to support this view point. However, luckily and importantly, we not only gained a promisingly novel biomarker for AMI diagnosis but also opened the first study on mechanism of miR-497 in human AMI.

In conclusion, circulating miR-497 might be a promising biomarker for AMI identification and there was high association between human miR-497 and acute myocardial infarction. It played fundamental roles in the further study on mechanism of miR-497 in human AMI.

## Figures and Tables

**Table 1:** Clinical characteristics of volunteers.

Characteristics	AMI group (*n* = 27)	Healthy group (*n* = 31)	*P* value
Age (years)	54.15 ± 11.34	51.21 ± 12.25	0.349
Gender (male/female)	20/7	16/15	0.079
WBC (×10^9^/L)	7.21 ± 2.21	6.29 ± 1.96	0.098
Cr (umol/L)	71 ± 35	58 ± 19	0.079
SBP (mmHg)	131 ± 25	119 ± 22	0.629
DBP (mmHg)	80 ± 13	74 ± 19	0.172
Glucose (mmol/L)	6.54 ± 1.58	6.72 ± 1.92	0.701
Hypertension, *n* (%)	12 (44%)	9 (29%)	0.223
DM, *n* (%)	4 (15%)	3 (10%)	0.549
TC (mmol/L)	4.55 ± 1.43	4.29 ± 1.11	0.342
LDL C (mmol/L)	2.72 ± 0.86	2.55 ± 0.64	0.393
TG (mmol/L)	1.44 ± 0.84	1.79 ± 1.22	0.215
HDL C (mmol/L)	1.21 ± 0.42	1.14 ± 0.37	0.503

Cr: creatinine; SBP: systolic blood pressure; DBP: diastolic blood pressure; DM: diabetes mellitus; TC: total cholesterol; LDL: low-density lipoprotein; TG: total triglyceride; HDL: high-density lipoprotein.

**Table 2:** miR-497 in human AMI patients and healthy adults.

	AMI (4 h)	AMI (8 h)	AMI (12 h)	AMI (24 h)	AMI (48 h)	AMI (72 h)	Healthy adults
Δct ± SD	−5.23 ± 0.67	−3.99 ± 1.47	−4.62 ± 1.35	−5.62 ± 1.68	−6.22 ± 0.86	−6.34 ± 1.15	−6.22 ± 1.58
*P* value	0.017	0.001	0.001	0.027	0.496	0.591	

**Table 3:** Areas under the ROC curve and predictive value of miR-497.

	AMI (4 h)	AMI (8 h)	AMI (12 h)	AMI (24 h)
2^−ΔΔCt^	2.18 ± 0.87	6.64 ± 4.13	3.91 ± 2.02	1.93 ± 0.99
95% CI	0.781–0.967	0.773–0.978	0.775–0.994	0.667–0.939
AUC	0.87	0.88	0.88	0.81
Cut-off point	1.52	1.55	1.61	1.37
Specificity (%)	90	94	95	84
Sensitivity (%)	81	82	85	80

2^−ΔΔCt^, relative expression of miR-497 (mean ± SD); 95% Cl, 95% confidence interval; AUC, area under the ROC curve.

## References

[1] White HD, Chew DP (2008). Acute myocardial infarction. *The Lancet*.

[2] Anderson JL, Adams CD, Antman EM (2007). ACC/AHA 2007 guidelines for the management of patients with unstable angina/non ST-elevation myocardial infarction: a report of the American College of Cardiology/American Heart Association Task Force on Practice Guidelines (Writing Committee to Revise the 2002 Guidelines for the Management of Patients With Unstable Angina/Non ST-Elevation Myocardial Infarction): developed in collaboration with. *Circulation*.

[3] Haaf P, Drexler B, Reichlin T (2012). High-sensitivity cardiac troponin in the distinction of acute myocardial infarction from acute cardiac noncoronary artery disease. *Circulation*.

[4] Alajez NM, Lenarduzzi M, Ito E (2011). MiR-218 suppresses nasopharyngeal cancer progression through downregulation of survivin and the SLIT2-ROBO1 pathway. *Cancer Research*.

[5] Fukushima Y, Nakanishi M, Nonogi H, Goto Y, Iwai N (2011). Assessment of plasma miRNAs in congestive heart failure. *Circulation Journal*.

[6] Wang GK, Zhu JQ, Zhang JT (2010). Circulating microRNA: a novel potential biomarker for early diagnosis of acute myocardial infarction in humans. *European Heart Journal*.

[7] Lewis BP, Burge CB, Bartel DP (2005). Conserved seed pairing, often flanked by adenosines, indicates that thousands of human genes are microRNA targets. *Cell*.

[8] Bartel DP (2009). MicroRNAs: target recognition and regulatory functions. *Cell*.

[9] Filipowicz W, Bhattacharyya SN, Sonenberg N (2008). Mechanisms of post-transcriptional regulation by microRNAs: are the answers in sight?. *Nature Reviews Genetics*.

[10] Cordes KR, Srivastava D, Ivey KN (2010). MicroRNAs in cardiac development. *Pediatric Cardiology*.

[11] Kim CW, Vo M, Kim HK (2012). Ectopic over-expression of tristetraprolin in human cancer cells promotes biogenesis of let-7 by down-regulation of Lin28. *Nucleic Acids Research*.

[12] Barringhaus KG, Zamore PD (2009). MicroRNAs regulating a change of heart. *Circulation*.

[13] Fichtlscherer S, De Rosa S, Fox H (2010). Circulating microRNAs in patients with coronary artery disease. *Circulation Research*.

[14] Tijsen AJ, Creemers EE, Moerland PD (2010). MiR423-5p as a circulating biomarker for heart failure. *Circulation Research*.

[15] Chim SSC, Shing TKF, Hung ECW (2008). Detection and characterization of placental microRNAs in maternal plasma. *Clinical Chemistry*.

[16] Margulies KB (2009). MicroRNAs as novel myocardial biomarkers. *Clinical Chemistry*.

[17] Sayed AS, Xia K, Yang TL, Peng J (2013). Circulating microRNAs: a potential role in diagnosis and prognosis of acute myocardial infarction. *Disease Markers*.

[18] Wang S, Li H, Wang J, Wang D (2013). Expression of microRNA-497 and its prognostic significance in human breast cancer. *Diagnostic Pathology*.

[19] Luo M, Shen D, Zhou X, Chen X, Wang W (2013). MicroRNA-497 is a potential prognostic marker in human cervical cancer and functions as a tumor suppressor by targeting the insulin-like growth factor 1 receptor. *Surgery*.

[20] Furuta M, Kozaki K, Tanimoto K (2013). The tumor-suppressive miR-497-195 cluster targets multiple cell-cycle regulators in hepatocellular carcinoma. *PLoS ONE*.

[21] Rink C, Khanna S (2011). MicroRNA in ischemic stroke etiology and pathology. *Physiological Genomics*.

[22] Yin KJ, Deng Z, Huang H (2010). miR-497 regulates neuronal death in mouse brain after transient focal cerebral ischemia. *Neurobiology of Disease*.

[23] Zhao S, Zhong Y, Jiang YH, Yi ZW (2013). Circulating microRNA expression in children with idiopathic short stature. *Chinese Journal of Contemporary Pediatrics*.

[24] Morrow DA, Cannon CP, Jesse RL (2007). National Academy of Clinical Biochemistry Laboratory Medicine Practice Guidelines: Clinical characteristics and utilization of biochemical markers in acute coronary syndromes. *Circulation*.

[25] Schmittgen TD, Livak KJ (2008). Analyzing real-time PCR data by the comparative CT method. *Nature Protocols*.

[26] Goren Y, Kushnir M, Zafrir B, Tabak S, Lewis BS, Amir O (2012). Serum levels of microRNAs in patients with heart failure. *European Journal of Heart Failure*.

[27] Turchinovich A, Weiz L, Langheinz A, Burwinkel B (2011). Characterization of extracellular circulating microRNA. *Nucleic Acids Research*.

[28] Tsui NBY, Ng EKO, Lo YMD (2002). Stability of endogenous and added RNA in blood specimens, serum, and plasma. *Clinical Chemistry*.

[29] de Guire V, Robitaille R, Tétreault N (2013). Circulating miRNAs as sensitive and specific biomarkers for the diagnosis and monitoring of human diseases: promises and challenges. *Clinical Biochemistry*.

[30] Fu Y, Yi Z, Wu X, Li J, Xu F (2011). Circulating microRNAs in patients with active pulmonary tuberculosis. *Journal of Clinical Microbiology*.

[31] Li LM, Hu ZB, Zhou ZX, Chen X, Liu FY, Zhang JF (2011). Serum microRNA profiles serve as novel biomarkers for HBV infection and diagnosis of HBV-positive hepatocarcinoma. *Cancer Research*.

[32] Cui L, Qi Y, Li H (2011). Serum microRNA expression profile distinguishes enterovirus 71 and coxsackievirus 16 infections in patients with hand-foot-and-mouth disease. *PLoS ONE*.

[33] Ji X, Takahashi R, Hiura Y, Hirokawa G, Fukushima Y, Iwai N (2009). Plasma miR-208 as a biomarker of myocardial injury. *Clinical Chemistry*.

[34] Oglesby IK, McElvaney NG, Greene CM (2010). MicroRNAs in inflammatory lung disease—master regulators or target practice?. *Respiratory Research*.

[35] Urbich C, Kuehbacher A, Dimmeler S (2008). Role of microRNAs in vascular diseases, inflammation, and angiogenesis. *Cardiovascular Research*.

